# Characteristics and evolution of knowledge innovation network in the Yangtze River Delta urban agglomeration——A case study of China National Knowledge Infrastructure

**DOI:** 10.1371/journal.pone.0283853

**Published:** 2023-04-21

**Authors:** Ya Gao, Lei Ye

**Affiliations:** 1 School of Geographic Science, Nantong University, Nantong, China; 2 School of Teacher Education, Nantong University, Nantong, China; 3 Jiangsu Yangtze River Economic Belt Research Institute, Nantong University, Nantong, China; Northeastern University (Shenyang China), CHINA

## Abstract

With the development of economic globalization, urban agglomerations have become growth poles and core areas of economic development. By building knowledge innovation networks in urban agglomerations, we can effectively improve the strength of inter-city knowledge innovation links and better realize the integrated and synergistic development of the region. This study selected core cities in the Yangtze River Delta urban agglomeration as the study area, constructed the knowledge innovation network based on inter-city dissertation cooperation data from 2010 to 2020, and analyzed the characteristics and evolution of its knowledge network by combining social network analysis and geospatial analysis. The research results show that: (1) with changes in policies and investment in scientific research and innovation, intra-regional thesis cooperation in the Yangtze River Delta urban agglomeration has been increasing and the scale of the knowledge innovation cooperation network is growing; (2) in addition to the core cities radiating innovation resources outward to drive the development of other node cities, other cities are continuously improving their own innovation capabilities, taking the initiative to strengthen knowledge innovation cooperation with core cities and enhancing their own position in the network; (3) there are no longer isolated cities within the Yangtze River Delta urban agglomeration, and a multi-core knowledge network structure centered on Shanghai, Nanjing, Hangzhou, and Suzhou has initially formed, but the network is still spatially heterogeneous; (4) there are still problems within the Yangtze River Delta urban agglomeration such as uneven development of knowledge innovation and low participation of peripheral cities, which need to be addressed jointly by all regions. The article concludes with some suggestions for countermeasures to provide a reference for the Yangtze River Delta urban agglomeration to continuously strengthen intra-regional knowledge cooperation in the future, enhance regional competitiveness, and ultimately achieve synergistic development among cities.

## 1. Introduction

In the context of the knowledge-based economy, China attaches great importance to science and technology innovation and development, emphasizing the need to implement the innovation-driven development strategy and adhere to the independent innovation path with Chinese characteristics. With the advancement of science and technology, the role of innovation in driving economic development is becoming increasingly significant and is gradually becoming an important element in improving the competitiveness of cities. Regional innovation systems have been extended based on national innovation systems, and the ever-richer theoretical and empirical research results have laid the foundation for conducting research on regional innovation networks [1]. Along with economic globalization and communication technology change, the external competitive environment has changed dramatically, and the cost and risk of innovation are greatly increased, so the innovation subjects are more and more dependent on the outside, and cooperation becomes the main way of knowledge innovation. As a form of spatial organization in the mature stage of regional development, urban agglomerations gather a large number of innovation resources and are the carriers of regional innovation activities. Extensive and in-depth knowledge and cultural exchanges are taking place among cities all the time, so the study of urban agglomerations and therefore urban agglomeration innovation networks has gradually become one of the hot topics in geography.

In the social development of the information age, the knowledge innovation system as a sub system of the innovation system is constantly recognized, and the innovation network is a way of expressing innovation research toward the network era. The issues related to inter-regional knowledge innovation cooperation have received the attention of many scholars worldwide. Foreign scholars have paid early attention to the content of research and innovation cooperation, and some scholars have used some data indicators to quantitatively study and analyze inter-regional research cooperation in the last century [2]; later scholars have used joint patent application data and thesis cooperation data to study the network situation of different objects, such as the network characteristics and evolution process of resource flow in the EU, the knowledge network of the music industry diversity, the network of research cooperation activities and the characteristics of innovation factor flows among Chinese provinces [3–5]. In recent years, some scholars have started to use social network analysis to study the connectivity of research networks and the network of citation performance of papers in the tourism industry [6,7]. After a brief review of domestic scholars’ research on knowledge innovation cooperation networks, it was found that they are mainly carried out from the following dimensions: (1) Different subject areas. Scholars have used co-authored papers to build knowledge cooperation networks and study structural characteristics and development patterns for different subject areas, such as competitive intelligence and management science [8,9]; while others have used networks as a basis to build a framework for regional network systems and innovation models to explore the impact of innovation networks on the performance of other industries [10,11]. In addition, some scholars used the network spatial method to analyze the population data, and then analyzed the spatial distribution characteristics of the population in Beijing [12]. (2) Different regional spaces. Scholars studying knowledge innovation networks in China have different regional boundaries. Some scholars have studied the knowledge network among cities in an urban agglomeration or an economic belt [13], while others consider prefecture-level cities as nodes to establish the knowledge innovation network in entire China [14]. Some scholars have studied the inter-provincial cooperation model and its characteristics based on the paper cooperation data of various provinces in China [15]; other scholars went beyond the limits of provincial administrative regions to select high-level paper cooperation networks among major cities for feature analysis [16]. (3) Different time spans. The research time span of scholars is also different, between 10 and 20 years for most of them, and then they were divided into corresponding time nodes to study the structure and evolution of knowledge networks in different regions [17–19].

At present, scholars at home and abroad have done a lot of research on knowledge innovation networks, and most of the studies at this stage focus on some specific fields, such as management science and competitive intelligence; some also focus on knowledge innovation networks between cities above the prefecture level or between provinces, but most of them use thesis cooperation data as the basis for constructing knowledge innovation networks, especially the case studies in the Guangdong-Hong Kong-Macao Greater Bay Area, and Beijing-Tianjin-Hebei region are the most numerous, while case studies of the Yangtze River Delta urban agglomeration are relatively few. Therefore, this paper takes the more economically developed Yangtze River Delta urban agglomeration as the research area, selects 26 core cities among them as the research objects, and uses social network analysis and geospatial analysis to study the regional knowledge innovation network with the help of dissertation cooperation data among cities, so as to dynamically grasp the spatial and temporal patterns and evolutionary characteristics of knowledge innovation cooperation among cities in the region, and provide decision-making references for enhancing the competitiveness of cities in the Yangtze River Delta urban agglomeration, the overall innovation capacity and regional collaborative development, which is of great significance for establishing multi-level science and technology innovation centers and improving the regional urban innovation system.

## 2. Materials and methods

### 2.1 Study area

The Yangtze River Delta urban agglomeration is one of the six largest urban agglomerations in the world. It is an important global base for advanced manufacturing and a world-class urban agglomeration with a global influence. Many cities within it are closely interconnected and are full of fierce competition. The 2019 Outline of the Yangtze River Delta Regional Integrated Development Plan issued by the State Council of the CPC Central Committee points out that the Yangtze River Delta region is one of the regions with the most active economic development, the highest degree of openness, and the strongest innovation capacity in China; it holds a pivotal strategic position in the overall national modernization and all-round opening pattern [20]. Although the land area of the Yangtze River Delta urban agglomeration accounts for only 3.7% of the country’s total land area, it has strong economic strength, abundant scientific and educational resources, and strong regional innovation capacity. Statistics show that the total economic volume of the Yangtze River Delta urban agglomeration accounts for approximately 25% of the country’s total economic volume. It has two comprehensive national science centers and more than 300 institutions of higher learning, and the annual expenditure on Research and Development(R&D) and the number of valid invention patents account for about 1/3 of the country. After several expansions, the planning scope of the Yangtze River Delta urban agglomeration now includes entire Shanghai, Jiangsu Province, Zhejiang Province, and Anhui Province. Considering the variability in the level of economic development and the allocation of innovation resources among cities in each province, this study selected 26 cities, including Shanghai, Nanjing, Wuxi, Suzhou, Hangzhou, Ningbo, Huzhou, Hefei and Wuhu, the core cities of the Yangtze River Delta urban agglomeration, based on the planning scope defined in the Yangtze River Delta Urban Agglomeration Development Plan adopted by the State Council meeting in 2016.

### 2.2 Data sources

At present, the main ways of knowledge interaction among universities, research institutes, and various enterprises in each city are joint patent application and multi-party cooperation in publishing papers, among which patents are the results of technological innovation and papers are the results of knowledge innovation, so the data of joint patent application and paper cooperation are important indicators reflecting the degree of regional innovation cooperation [21]. Because the data on joint patent applications between cities in the Yangtze River Delta region are relatively lacking and difficult to query, this study selects inter-city dissertation cooperation data as an indicator of the degree of inter-city innovation cooperation, and uses 26 cities as network nodes to construct a knowledge innovation network for the Yangtze River Delta urban agglomeration. The China National Knowledge Infrastructure (CNKI) provides a variety of databases, including source databases, foreign language databases, economic databases and educational databases, which contain many articles such as journals, master’s degree theses, full text of important newspapers and conferences, and each database provides a search function to obtain data. Therefore, this paper retrieves the dissertation cooperation data through CNKI database, specifies the study period as 2010–2020, adds the author unit field in the advanced search page, selects author unit = City A AND author unit = City B to retrieve the number of dissertation cooperation between two cities and constructs the dissertation cooperation data matrix between two cities accordingly.

In addition, the data on the administrative divisions of China’s prefecture-level cities used to produce the thematic maps came from the Data Center for Resources and Environmental Sciences of the Chinese Academy of Sciences(RESDC). Some scholars used the architectural data, meteorological data and GF1 images provided by this database to study the influence of different urban forms on urban surface temperature in Beijing and Guangzhou [22–24]. The above articles provide good data source and method basis for the work of map editing and data analysis in this paper.

### 2.3 Methods

There are two main research methods in this paper, which are social network analysis and geospatial analysis. Firstly, we use social network analysis to obtain the network density, network centrality and other data results. Then the visualization of network organization structure and spatial structure is carried out by geospatial analysis method.

#### 2.3.1 Social network analysis

As a new research method in the field of social sciences, social network analysis focuses more on the overall structure of the network, with unique innovations in data collection and methodology, and tends to represent results in the form of visual diagrams and social network structures [25]. Social network analysis can analyze networks from various perspectives through centrality analysis and cohesive subgroup analysis, thus revealing the overall characteristics of the network and the relationships between nodes within the network from different perspectives; therefore, it is widely used in the study of network structure at different scales in various subjects such as mathematics and statistics. In this study, we used network density analysis and network centrality analysis to study network relationships and their evolution through knowledge cooperation among cities in the Yangtze River Delta urban agglomeration.

(1) Network density

Network density indicates the sparseness of a network, which can intuitively reflect the closeness of interconnections between various nodes in the network. Domestic and foreign scholars have shown that a high-density network can improve knowledge transfer and innovation and promote the exchange of information and knowledge diffusion within the network [26]. In this study, we examine the intra-regional knowledge innovation cooperation network in the Yangtze River Delta urban agglomeration, where a higher network density represents a stronger inter-city knowledge innovation linkage. The calculation formula is as follows:

D=m/[n×(n−1)] (1)

where *D* is the network density, *m* is the actual number of connections between nodes in the network, and *n* is the number of nodes in the network [27].

(2) Network centrality

Network centrality analysis is often used to analyze network nodes in social network analysis, and can reflect the position of network nodes in the network; it mainly contains the three indicators of point degree centrality, betweenness centrality, and proximity centrality.

Point degree centrality refers to the number of connections a node has with other nodes in the network and is an indicator of the importance of a city in the network. In the knowledge network of the Yangtze River Delta urban agglomeration, if a node city has the highest point degree centrality, it also has the highest status in the network and can establish connections with more nodes, that is, it is considered to be the central city in the network with many best resources and other advantages. The calculation formula is as follows:

$$C_{D}(i)=d(i)/(n-1)$$ (2)

where *C*_*D*_(*i*) is the value of a node’s point centrality, *d*(*i*) is the number of nodes in the network with which the node is associated, and N is the number of nodes in the network [27].

Betweenness centrality refers to the ability of a node in the network to act as an intermediary, enabling any two other nodes connected to it to communicate with each other; that is, the degree of dependence of other cities on that node city. In the knowledge network of the Yangtze River Delta urban agglomeration, if a city has the largest betweenness centrality value, it means that the other cities are most dependent on this city, so that city has more control over the other cities and also has more power to allocate resources. The calculation formula is as follows:

$$C_{b}(i)=\frac{\sum g_{jk}(i)/g_{jk}}{\left(n-1)(n-2\right)}$$ (3)

where *C*_*b*_(*i*) is the value of the betweenness centrality of nodes, *g*_*jk*_ is the number of shortest paths between nodes *j* and *k*, and *g*_*jk*_(*i*) is the number of shortest paths generated by node *j* to node *k* through node *i* [27].

Proximity centrality refers to the degree to which a node in a network is not controlled or influenced by the other nodes. If a node in the network is connected to multiple nodes through a short path, the proximity centrality of the node is high [28]. In the knowledge network of the Yangtze River Delta urban agglomeration, the smaller the value of proximity centrality, the more centrally located is the node city in the network. The calculation formula is as follows:

$$C_{C}(i)=\frac{\left(n-1\right)}{\sum_{j=1}^{n}d(i,j)}$$ (4)

where *C*_*C*_(*i*) is the proximity centrality of node *i*, *d*(*i*, *j*) is the number of shortest paths between nodes *i* and *j* in the network, and *n* is the number of network nodes [27].

#### 2.3.2 Geospatial analysis

ArcGIS software was used to visualize the knowledge innovation network of the Yangtze River Delta urban agglomeration in geographic space. The specific operation steps are as follows: (1) create a layer of the study area using ArcGIS software and draw lines representing the links between cities to build a knowledge innovation network of the Yangtze River Delta urban agglomeration; (2) modify the color and thickness of the lines in the network through the graded color setting method, using lines of different colors and thickness to indicate the strength of knowledge links between cities; (3) add map elements such as a compass and scale to draw a thematic map.

## 3. Results

### 3.1 Evolution of the general characteristics of knowledge networks

This study is based on the 2010–2020 Yangtze River Delta urban agglomeration dissertation cooperation data collected in the CNKI database, selects the data from 2010, 2015, and 2020 to construct an inter-city dissertation cooperation matrix, and uses UCINET software to calculate the overall network density. The results are shown in Table 1. By analyzing the dissertation cooperation data during the study period, the total number of dissertations in the Yangtze River Delta urban agglomeration shows an increase, from 8,881 in 2010 to 10,843 in 2020, which is mainly dominated by the external dissertation cooperation of Shanghai and Nanjing. Over time, the number of collaborative papers from several cities in Anhui Province steadily increased, indicating that they have received abundant resources for research and innovation, including talent and technology, because of their participation in the integrated development of the Yangtze River Delta region. In the constructed completed dissertation collaboration matrix, if any two cities for which dissertation collaboration data exist are said to be one city group, then there are 325 city groups in the study area. There were 206 city groups that cooperated in 2010, 218 city groups that cooperated in 2015, and 252 in 2020. The results suggest that there are no longer isolated cities in the study area, but there are still a small number of cities that have not generated innovative collaboration and have failed to achieve full coverage of knowledge networks in the region. The calculation showed that the knowledge innovation network density of the Yangtze River Delta urban agglomeration was 0.6123 in 2010, 0.6708 in 2015, and 0.7754 in 2020. In 2015, the network density increased by 9.5% compared with that in 2010; in 2020, the network density increased by 15.6% compared with that in 2015, indicating an accelerated growth rate of network density, demonstrating the increasing scientific productivity of the cities in the Yangtze River Delta urban agglomeration, the gradually increasing capacity for internal innovation and cooperation, and the growing maturity and stability of knowledge links.

**Table 1:** Data on the overall characteristics of knowledge networks in the Yangtze River Delta urban agglomeration.

	2010	2015	2020
Number of thesis collaborations	8881	9721	10843
Number of city groups	206	218	252
Knowledge network density	0.6123	0.6708	0.7754

The thesis collaboration data show that Shanghai leads the way in the number of thesis collaborations with other cities and is the top city in the knowledge network of the Yangtze River Delta urban agglomeration, followed by the capitals of Jiangsu and Zhejiang provinces, and some economically strong cities. Most cities in Anhui Province have fewer thesis collaborations, but the number of thesis collaborations has been increasing as they gradually join the Yangtze River Delta urban agglomeration. Shanghai is a National Innovation Demonstration Zone, and as the center of the urban agglomeration, its economic strength, research and innovation resources, and education levels are higher than those of other cities. Nanjing, which has the most dense cluster of universities in Jiangsu Province, also has a clear advantage. Both cities are firmly in the top two in terms of cooperation with other cities. Shanghai and Nanjing are closely linked with each other and have a much higher level of cooperation in knowledge and innovation than other cities, which has a certain driving effect on the technological innovation of the surrounding cities.

#### 3.2 Evolution of knowledge network organization structure

The dissertation cooperation data of each city in the Yangtze River Delta urban agglomeration in 2010, 2015, and 2020 were selected, a symmetric matrix in the UCINET software was established, and the social network analysis method was used to analyze the organizational structure evolution characteristics of the regional knowledge network.

The software was used to calculate the centrality of knowledge network points for the Yangtze River Delta urban agglomeration in 2010, 2015, and 2020 (as shown in S1 Table), and analysis of the data leads to the following conclusions: (1) Nanjing, Shanghai, Suzhou, Wuxi, Changzhou, and Hangzhou ranked steadily among the top six and are in an important position in the network, indicating that the development of science and technology innovation in the Yangtze River Delta urban agglomeration is no longer influenced by a single city, but a small subgroup of cities leading the way. Among them, Nanjing has a large number of colleges and universities, has the advantage of talent science and education, announced the construction of an innovative city as early as 2006, became the only pilot city of science and technology reform in 2009, and kept forming the mechanism of cooperation between enterprises and universities and research institutes through the reform of science and technology system, and gradually transformed the advantage of science and education into the advantage of innovation. Therefore, Nanjing has a higher ability to publish academic papers than other cities, and is firmly in the first place in terms of point degree centrality, and its point degree centrality value is increasing, indicating that Nanjing has increasing knowledge and innovation resources and increasing scientific research ability. (2) The rankings of Hangzhou, Ningbo, Huzhou, and Zhoushan in Zhejiang Province gradually increased from 2010 to 2020, while the rankings of Jiaxing and Shaoxing remained stable and increased over time. This is because since 2017, Shanghai and Zhejiang Province have been working together to create the Hangzhou Bay Greater Bay Area, providing strategic planning for the development of high-tech industries, which has led to the continuous improvement of innovation and entrepreneurship support policies and the gradual completion of service systems in some cities in Zhejiang Province, and the initial construction of an industrial innovation center for the digital economy, which has led to the strengthening of inter-city knowledge and innovation ties. (3) Except for the provincial capital city of Hefei, the other cities in Anhui Province are at the tail end of the point degree centrality ranking and the edge of the knowledge innovation network, but their point degree centrality values gradually increase, indicating that each city has, to a certain extent, improved its knowledge creation capability and actively cooperated with the outside world in innovation.

**Fig 1:**
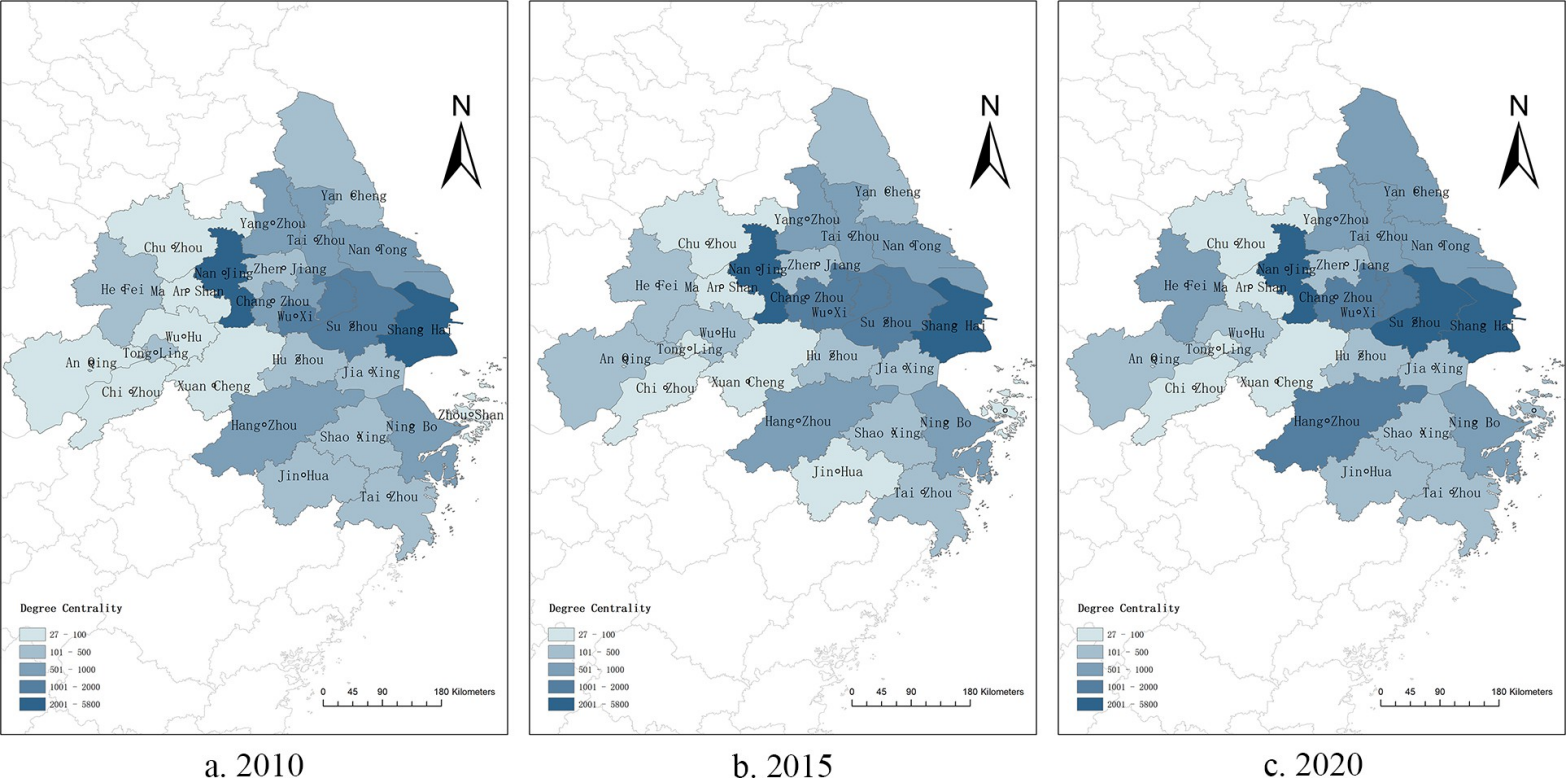
Point degree centrality of knowledge innovation network in Yangtze River Delta urban agglomeration in 2010, 2015 and 2020.

The point degree centrality data were plotted using ArcGIS showing the change in the point degree centrality of each city in the Yangtze River Delta urban agglomeration over time. As shown in Fig 1, except Ma’anshan, Xuancheng, and Chizhou, the point degree centrality ranks of the remaining cities have changed significantly, especially in Suzhou, Yancheng, Hangzhou, and Hefei, which indicates that the allocation of innovation resources in the Yangtze River Delta urban agglomeration has continued to rationalize, scientific research cooperation has increased, and knowledge links between the cities have become closer, led by the four cities of Shanghai, Nanjing, Hangzhou, and Hefei.

From the above analysis, it can be seen that the larger the value of betweenness centrality, the stronger the city’s ability to control resources and the more dependent the rest of the city is on it. The larger the value of proximity centrality, the more the city is controlled and influenced by other cities and is at the edge of the network. Therefore, in this study, we calculated the betweenness centrality and proximity centrality (as shown in S2 and S3 Tables). After comparative analyzed, the following conclusions can be drawn: (1) Shanghai, Nanjing, Suzhou, and Hangzhou all maintain a high level of betweenness centrality, and have long been the “middlemen” of the network, controlling most of the knowledge and innovation cooperation resources of the Yangtze River Delta urban agglomeration and enabling knowledge and innovation cooperation and linkages among other cities. By contrast, these four cities remain in the top four having the least proximity centrality, meaning that they are the least dependent on other cities, reflecting their strong research and knowledge innovation linkage capabilities. (2) Whether it is betweenness centrality or proximity centrality, Chizhou, Xuancheng, and Zhoushan always rank last, indicating that these three cities have the least control and influence on the scientific research development and knowledge innovation cooperation of other cities and are the most dependent on the knowledge resources of other cities, which also reflects from the perspective that the knowledge innovation cooperation capacity of these three cities is the weakest in the Yangtze River Delta urban agglomeration. (3) With the construction of the Hangzhou Bay Area, cities such as Hangzhou, Ningbo, and Jiaxing in Zhejiang Province have continuously improved the betweenness centrality of the network under the leadership of Shanghai, and proximity centrality has been decreasing. Similarly, cities, such as Hefei, Wuhu, Changzhou, and Nantong, show that most cities in the Yangtze River Delta urban agglomeration are continuously improving their research and innovation capabilities and their knowledge cooperation capabilities with other cities. Their dependence on the knowledge resources of the central city of the network is gradually decreasing, which also indicates their increasing ability to generate knowledge links with other surrounding cities.

**Fig 2:**
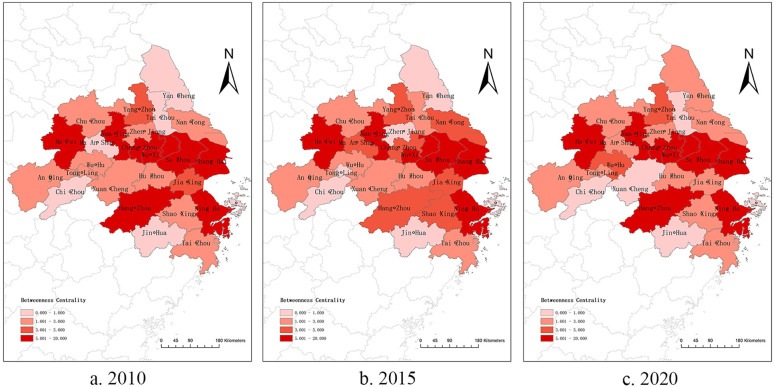
Betweenness centrality of knowledge innovation networks in the Yangtze River Delta urban agglomeration in 2010, 2015 and 2020.

**Fig 3:**
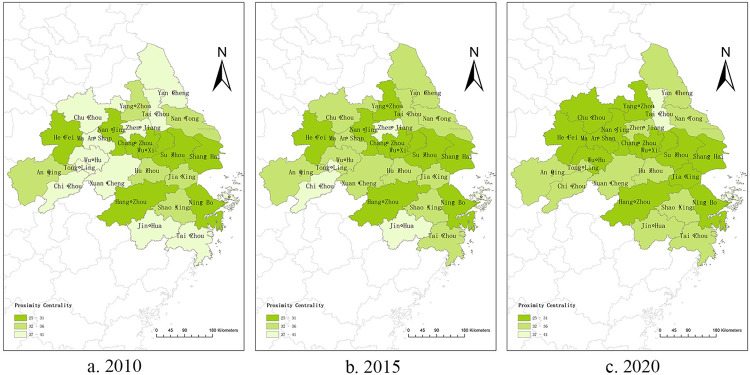
Proximity centrality of knowledge innovation networks in the Yangtze River Delta urban agglomeration in 2010, 2015 and 2020.

The changes in betweenness centrality and proximity centrality were plotted using ArcGIS. The betweenness centrality of most cities in the eastern and northern parts of the Yangtze River Delta urban agglomeration increases significantly, while proximity centrality generally decreases (as shown in Figs 2 and 3). From 2010 to 2020, the network intermediate centrality potential of the Yangtze River Delta urban agglomeration decreased from 4.99% to 1.25%, and the network proximity potential decreased from 55.93% to 35.86%, indicating a stable decreasing state. This indicates that the knowledge innovation network of the Yangtze River Delta urban agglomeration is gradually improving, and the knowledge innovation cooperation capability within the urban agglomeration is gradually enhanced.

### 3.3 Evolution of the spatial landscape of knowledge networks

**Fig 4:**
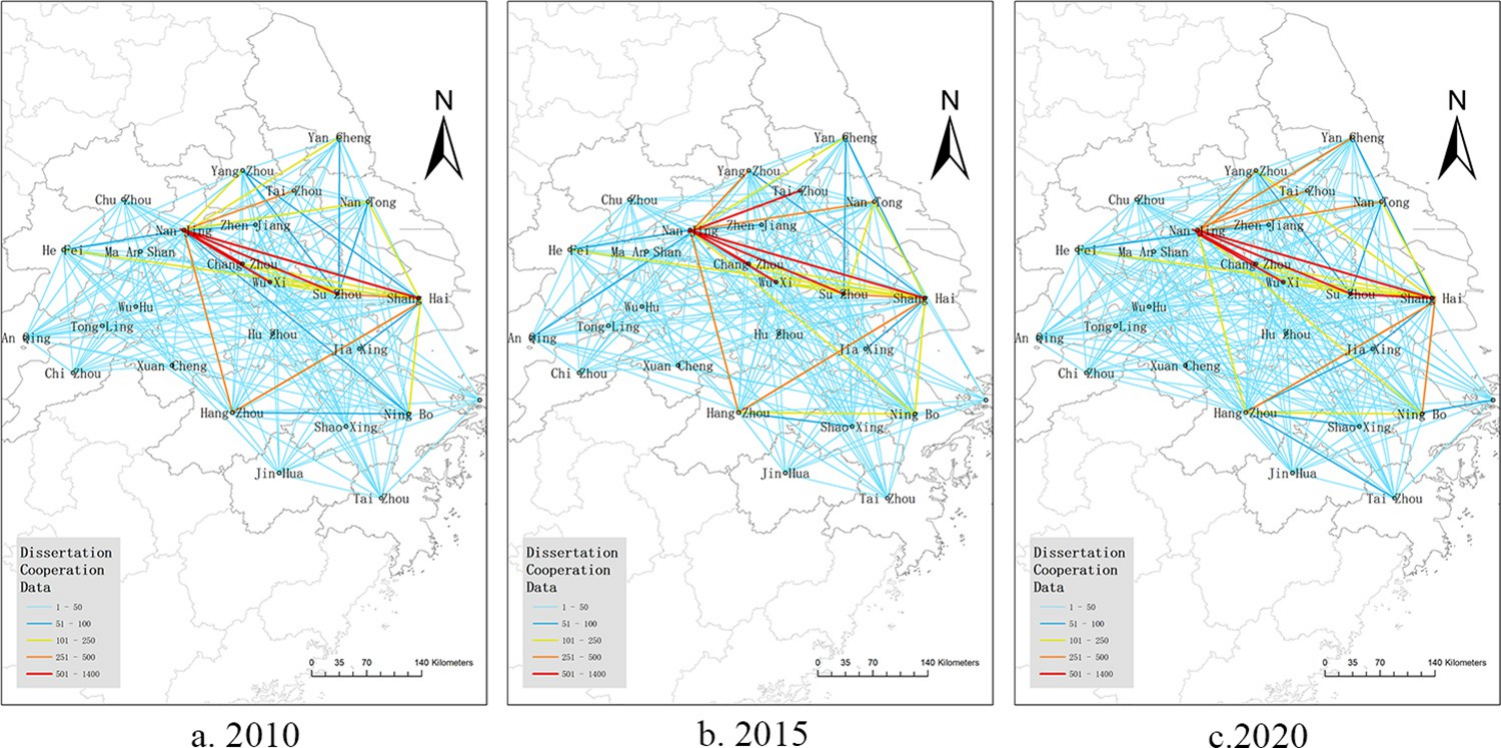
Knowledge innovation network map of Yangtze River Delta urban agglomeration in 2010, 2015 and 2020.

To analyze the evolutionary characteristics of the spatial pattern of the knowledge innovation network of the Yangtze River Delta urban agglomeration, the dissertation cooperation data for 2010, 2015, and 2020 were visualized using ArcGIS software to draw a spatial map of the knowledge network. Fig 4 shows the increase in the number of dissertation collaborations between 2010 and 2020 corresponds to an increase in the number of links between nodes in the network, demonstrating the increasing overall size of the network and the closer knowledge links within the entire Yangtze River Delta urban agglomeration.

Overall, the knowledge innovation cooperation network of the Yangtze River Delta urban agglomeration exhibits an intricate and complex spatial distribution pattern. The cities in the central and eastern parts of the region form strong innovation links, whereas those in the western and more peripheral parts of the region are weaker, showing a dense spatial structure in the east and sparse in the west. The overall innovation network is a “core-periphery” structure. The cities of Shanghai, Nanjing, and Hangzhou are connected to form a relatively stable triangle and radiate outward to create innovative cooperation with other cities. Shanghai is at the absolute core of the dissertation cooperation network in the Yangtze River Delta urban agglomeration, and Nanjing and Hangzhou play a role in the communication and connection of the entire network. Specifically, the evolution of the spatial pattern of knowledge innovation networks is characterized by the following features:

#### 3.3.1 There was significant spatial heterogeneity

Fig 4 shows that, from 2010 to 2020, there is a significant unevenness in the knowledge innovation network, with innovation linkages of medium and higher intensity mainly distributed in the eastern part of the urban agglomeration and dominated by knowledge innovation cooperation between cities within Jiangsu and Zhejiang provinces, with relatively weak innovation cooperation between provinces. This shows that administrative divisions and regional policy regimes have a deterrent effect on inter-city innovation cooperation. Over time, the degree of innovation linkages between cities in the eastern part of the Yangtze River Delta urban agglomeration has increased, particularly noticeable in 2020, but weaker innovation cooperation is still distributed in the western part of the region, that is, some cities in Anhui Province, joined the Yangtze River Delta urban agglomeration relatively late and had a lower level of participation in the innovation network.

#### 3.3.2 Gradually evolving into a polycentric core-periphery structure

**Fig 5:**
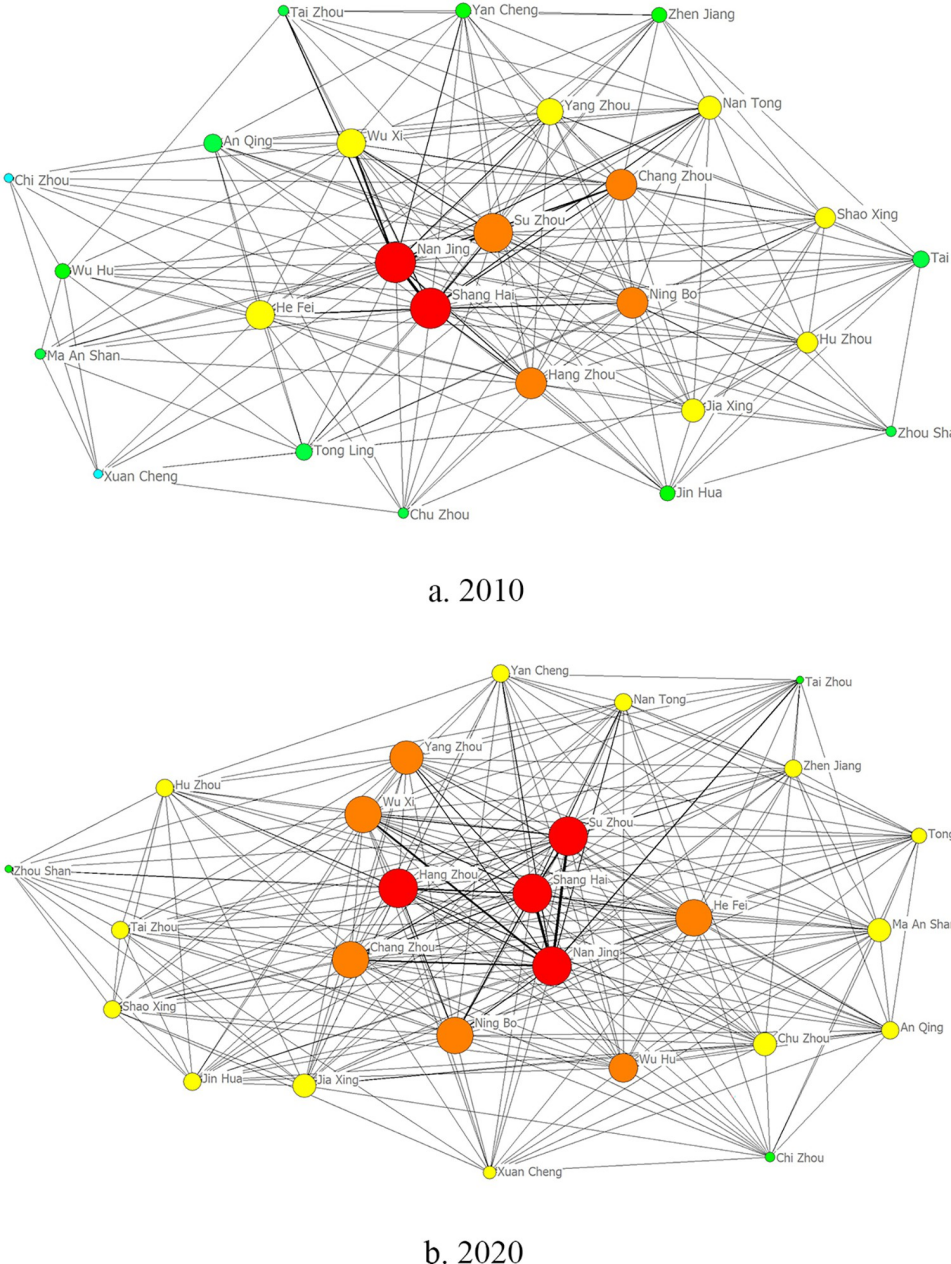
Ranking of node cities in the Yangtze River Delta urban agglomeration in 2010 and 2020.

As shown in Fig 4, the hierarchical phenomenon of the knowledge innovation network of the Yangtze River Delta urban agglomeration in 2010 is relatively obvious, showing a spatially dispersed pattern of radiation centered on Shanghai and Nanjing, with relatively weak links between other nodes in the region and fragmented distribution. In 2015, the network density in the region increased, and Hangzhou City, Zhejiang Province and Hefei City, Anhui Province strengthened their cooperation with other cities in the province and became secondary core node cities in the network. In 2020, the inter-city network between cities in the Yangtze River Delta urban agglomeration further increases in size, and innovation links become closer, with cities in southern Jiangsu Province such as Nanjing, Suzhou, and Wuxi strengthening innovation cooperation with cities in northern Jiangsu Province such as Yangzhou and Yancheng, and with most cities in Anhui Province already having established innovation links; Hangzhou and Ningbo in Zhejiang Province also strengthen innovation links with Shanghai, forming a triangular framework and radiating outward, and cooperation with edge cities such as Zhoushan and Huzhou has gradually increased. However, the knowledge innovation network diagram does not clearly reflect hierarchical evolution of node cities, so the absolute degree centrality of cities, that is, the number of cities with which a city has a direct connection, is calculated by UCINET software and visualized to further understand the evolution of node cities. The data for 2010 and 2020 were selected for calculation, and the absolute degree centrality was divided into five tiers, with D = 25 as the first tier, 20≤D≤24 as the second tier, 15≤D≤19 as the third tier, and 10≤D<15 and D<10 as the fourth and fifth tiers. The NetDraw function was used to plot the node evolution of the Yangtze River Delta urban agglomeration, as shown in Fig 5.

The larger the absolute degree centrality of each city, the larger the nodes in the diagram, which are proportional, and the lines between the nodes represent the cities that generate knowledge and innovation links. The red, orange, yellow, green, and blue nodes represent tiers one to five, respectively, indicating cities in core, sub-core, general, and edge city status (where tiers four and five are all edge cities), respectively. It can be seen from Fig 5 that in 2010, the innovation links between cities were weak and the network was sparse. Nanjing and Shanghai were the core cities in the network, while Suzhou, Changzhou, Hangzhou, and Ningbo were the secondary core cities. Other cities were scattered and the participation in knowledge innovation cooperation in the network was low. Gradually, the knowledge innovation connection between cities has become increasingly close, and the spatial structure of the knowledge innovation network of the Yangtze River Delta urban agglomeration has become more complex. By 2020, Suzhou and Hangzhou will also become the core cities in the network, and together with Shanghai and Nanjing, they will drive the neighboring cities to innovation development; Hefei, Wuhu, Yangzhou, and Wuxi will become secondary cores; Yancheng, Zhenjiang, Taizhou and Chuzhou will rise from peripheral cities to ordinary node cities; only Xuancheng, Taizhou, and Zhoushan will remain as peripheral cities in the network, and their innovation cooperation with other cities will be limited, showing a multi-center and multi-level core-peripheral structure.

#### 3.3.3 Intra-regional knowledge cooperation is increasing and the intensity of innovation links is growing

In 2010, the knowledge innovation network was mainly built on the cooperation of papers from Shanghai, Hangzhou, and some southern cities in Jiangsu Province, because there are many famous national historical and cultural cities in this area, as well as a large number of institutions of higher learning and scientific research institutions. It has a rich historical, cultural, scientific, and educational talent resource. The number of dissertation collaborations in most cities in Jiangsu and Zhejiang provinces increased in 2015, and network density rose significantly. The Regional Plan for the Yangtze River Delta Region released in 2010 proposed that the region should innovate the mode of cooperation between industry, academia, and research; establish a mechanism for co-creation of results, information sharing, and benefit sharing among all parts of the subjects; and encourage multiple forms of cooperation to promote open sharing of scientific and technological resources. As the core cities of the Yangtze River Delta urban agglomeration at the time, knowledge innovation ties were bound to be gradually strengthened. In the knowledge innovation network in 2020, Shanghai is more closely related to Jiangsu and Zhejiang provinces, and the network density in Anhui Province has increased. Since 2016, Anhui has continuously participated in the integrated development of the Yangtze River Delta. By 2019, all cities in Anhui Province had joined the Yangtze River Delta urban agglomeration, and in its 13th Five-Year Plan, it was proposed to strengthen investment in innovation. Through the establishment of a comprehensive national science center in Hefei, a government management mechanism that conforms to the law of innovation and a new mechanism conducive to talent incentives and agglomeration, the province’s scientific research, and innovation capabilities will be strengthened, and the integration of the Yangtze River Delta urban agglomeration will be accelerated to participate in innovation cooperation. With the passage of time the development of the integration of the Yangtze River Delta, the knowledge innovation cooperation of the Yangtze River Delta urban agglomeration is no longer mainly within the province. The knowledge innovation cooperation between provinces has increased, and the influence of different policies and administrative divisions has gradually decreased. The innovation linkages, between cities in a region, are becoming more frequent.

## 4. Conclusion and discussion

### 4.1 Conclusion

The innovation of knowledge and technology is inseparable from the construction of urban agglomeration innovation network, and the urban agglomeration innovation network is also an important part of the national innovation system, so it is important to study the knowledge innovation network of the Yangtze River Delta urban agglomeration. In this study, 26 core cities in the Yangtze River Delta urban agglomeration were considered as the research object, and a knowledge innovation cooperation network was constructed using inter-city dissertation cooperation data. The analysis draws the following conclusions: (1) The construction of the knowledge innovation network of the Yangtze River Delta urban agglomeration has been achieved to cover all cities, and Shanghai, Nanjing, Suzhou and Hangzhou firmly occupy the core position of the network with the advantages of many comprehensive institutions of higher education and science and technology innovation industries, maintain close knowledge cooperation with other cities, and drive the development of science and technology innovation in other node cities. (2) The knowledge innovation network of the Yangtze River Delta urban agglomeration has obvious spatial heterogeneity, but with the changes in regional policies and research investment, the knowledge innovation network is in the process of continuous improvement, the knowledge links between cities in the region are strengthening, and some sub-core cities are strengthening their own capacities and have become less dependent on the core cities. (3) At present, the knowledge innovation network of the Yangtze River Delta urban agglomeration has formed a polycentric core-periphery structure, so the development and changes of the network are no longer controlled by one or two cities, but will be influenced by small subgroups of cities. (4) Although the Yangtze River Delta urban agglomeration is economically developed and in a leading position nationwide, with high inter-city connectivity, there are still problems, such as regional differences in knowledge innovation cooperation and uneven distribution of innovation resources, making it difficult for the edge cities of the network to develop knowledge innovation and improve their research capacity. For example, Chizhou, Xuancheng, Zhoushan, and other peripheral cities have fewer knowledge resources and are highly dependent on other cities, which limits their research capacity.

### 4.2 Discussion

The research on knowledge innovation network of Yangtze River Delta urban agglomeration in this paper has. Firstly, most of the previous studies on knowledge innovation networks in urban agglomerations are based on the cooperation of papers in a specific discipline, while this paper selects all the cooperative papers among cities and is not limited to a certain field, so the data are more comprehensive. Secondly, this paper not only uses social network analysis to analyze the organizational structure characteristics of urban agglomeration networks, but also uses ArcGIS visualization technology to analyze the spatial structure of networks. Finally, the research data and methods adopted in this paper are universally applicable, which are of great significance to the future exploration of knowledge innovation networks in other urban agglomerations and even national and global cities.

At present, under the leadership of national policies, the integrated development of Yangtze River Delta region has risen to become a national strategy, and the new round of scientific and technological revolution and industrial change in the world as well as the optimization and upgrading of China’s economy have provided a good external environment for the integrated development of Yangtze River Delta, so the Yangtze River Delta urban agglomeration has taken the lead in the key stage of transformation and upgrading, innovation and development, and inter-city knowledge cooperation has become an important form of innovation. Then by constructing the knowledge innovation network of the Yangtze River Delta urban agglomeration, we can understand the basic characteristics of urban knowledge cooperation, dynamically analyze the changes and laws of the network structure, and better guide the high-quality development of knowledge cooperation in the Yangtze River Delta or other urban agglomerations from the policy level. In order to improve the overall innovation capability and city competitiveness of the Yangtze River Delta urban agglomeration, strengthen the cooperation and exchange of knowledge innovation within the region, and promote the integrated development of the Yangtze River Delta and the construction of the innovation system of the urban agglomeration, this paper puts forward the following countermeasure suggestions. First, the government departments of each province and city should implement an innovation-driven development strategy, speed up the construction of knowledge innovation cooperation platforms, make joint efforts to build a “knowledge innovation community” in the Yangtze River Delta, and rely on digitalization and information technology to integrate various resources and promote the exchange and sharing of knowledge, information, and innovation elements. Second, the government needs to formulate innovation cooperation incentive policies to promote cross-regional coordination between innovation and industry chains, so that cities can take advantage of various innovation resources agglomerations, improve the division of urban industries, and promote the cross-regional alliance of universities, enterprises, and scientific research institutions. Third, government departments should increase support for edge cities, enhance the knowledge innovation capacity of edge cities through talent attraction policies, subsidies for research funding and university-enterprise alliances, promote good interaction and cooperation between edge cities and core cities, and improve the knowledge innovation network of the Yangtze River Delta urban agglomeration. Finally, in the context of the existing spatial pattern of “one core, five circles, and four belts” in the Yangtze River Delta urban agglomeration, the spatial layout of innovation resources will be optimized, and the formation of innovation functional areas and more knowledge network city nodes will be promoted. The core city of Shanghai and several regional centers cities will play the role of a radiation drive to enhance the overall innovation capacity of the region.

Since this paper only selects the paper cooperation data as an indicator of city knowledge innovation cooperation and fails to use other data such as joint patent applications as support, and the paper cooperation data is obtained through CNKI search, which fails to take into account other paper databases and the cooperation of authors from three or more cities in publishing articles, there are certain limitations, which is the direction of future efforts to improve the accuracy of research data as well as the depth of article research.

## Supporting information

S1 Table Point degree centrality of knowledge innovation network in Yangtze River Delta urban agglomeration. (PDF)

S2 Table Betweenness centrality of knowledge innovation networks in the Yangtze River Delta urban agglomeration. (PDF)

S3 Table Proximity centrality of knowledge innovation networks in the Yangtze River Delta urban agglomeration. (PDF)
