# Supplementary material for: Characteristics and evolution of knowledge innovation network in the Yangtze River Delta urban agglomeration——A case study of China National Knowledge Infrastructure
Source: PLoS One. 2023 Apr 21;18(4):e0283853. doi: 10.1371/journal.pone.0283853 (PMC10121040; doi:10.1371/journal.pone.0283853)
Supplement: S1 Table — (PDF) [file pone.0283853.s001.pdf]

S1 Table.

Ranking	City	2010	City	2015	City	2020
1	Nan Jing	5255	Nan Jing	5641	Nan Jing	5724
2	Shang Hai	3152	Shang Hai	3260	Shang Hai	3603
3	Su Zhou	1849	Su Zhou	1906	Su Zhou	2461
4	Wu Xi	1284	Wu Xi	1341	Wu Xi	1390
5	Chang Zhou	935	Chang Zhou	1071	Hang Zhou	1126
6	Hang Zhou	875	Hang Zhou	943	Chang Zhou	1090
7	Nan Tong	676	Tai Zhou	777	Ning Bo	972
8	Ning Bo	572	Ning Bo	728	Nan Tong	730
9	Yang Zhou	569	Nan Tong	681	Yang Zhou	704
10	Tai Zhou	501	Yang Zhou	567	Tai Zhou	593
11	He Fei	356	Yan Cheng	457	He Fei	550
12	Yan Cheng	337	He Fei	424	Yan Cheng	514
13	Jia Xing	201	Jia Xing	215	Jia Xing	279
14	Shao Xing	159	Shao Xing	176	Shao Xing	241
15	Zhen Jiang	152	Tai Zhou	172	Hu Zhou	238
16	Tai Zhou	141	Hu Zhou	161	Zhen Jiang	211
17	Hu Zhou	133	Zhen Jiang	151	Tai Zhou	198
18	Jin Hua	114	An Qing	134	Zhou Shan	195
19	Tong Ling	110	Wu Hu	101	Jin Hua	154
20	An Qing	94	Chu Zhou	97	An Qing	152
21	Wu Hu	77	Tong Ling	91	Wu Hu	148
22	Zhou Shan	64	Jin Hua	81	Chu Zhou	100
23	Chu Zhou	49	Ma An Shan	78	Ma An Shan	97
24	Ma An Shan	46	Zhou Shan	77	Tong Ling	88
25	Chi Zhou	34	Xuan Cheng	75	Xuan Cheng	68
26	Xuan Cheng	27	Chi Zhou	37	Chi Zhou	60
