# Supplementary material for: Characteristics and evolution of knowledge innovation network in the Yangtze River Delta urban agglomeration——A case study of China National Knowledge Infrastructure
Source: PLoS One. 2023 Apr 21;18(4):e0283853. doi: 10.1371/journal.pone.0283853 (PMC10121040; doi:10.1371/journal.pone.0283853)
Supplement: S2 Table — (PDF) [file pone.0283853.s002.pdf]

S2. Table.

Ranking	City	2010	City	2015	City	2020
1	Shang Hai	19.249	Shang Hai	14.133	Shang Hai	6.424
2	Nan Jing	19.249	Nan Jing	14.133	Nan Jing	6.424
3	Su Zhou	15.435	Su Zhou	10.975	Su Zhou	6.424
4	Chang Zhou	8.793	Ning Bo	9.163	Hang Zhou	6.424
5	He Fei	8.560	Wu Xi	6.296	Chang Zhou	5.636
6	Hang Zhou	7.925	He Fei	5.500	Wu Xi	5.342
7	Wu Xi	7.248	Hang Zhou	4.978	Ning Bo	5.192
8	Ning Bo	7.016	Yang Zhou	4.975	He Fei	5.110
9	Yang Zhou	4.840	Shao Xing	4.839	Yang Zhou	4.437
10	Jia Xing	4.339	Nan Tong	4.721	Wu Hu	3.042
11	Hu Zhou	2.645	Chang Zhou	3.904	Jia Xing	2.480
12	An Qing	2.545	Jia Xing	3.720	Chu Zhou	2.283
13	Tong Ling	2.509	Ma An Shan	3.284	Ma An Shan	2.185
14	Nan Tong	2.468	An Qing	2.533	Shao Xing	1.341
15	Wu Hu	2.452	Wu Hu	2.373	Tai Zhou	1.242
16	Shao Xing	1.981	Hu Zhou	2.114	Yan Cheng	1.164
17	Xuan Cheng	1.450	Tai Zhou	1.837	Nan Tong	1.158
18	Tai Zhou	1.225	Xuan Cheng	1.663	Zhen Jiang	1.117
19	Zhen Jiang	1.077	Chu Zhou	1.556	Tong Ling	1.113
20	Chu Zhou	1.037	Tai Zhou	1.222	An Qing	1.049
21	Ma An Shan	0.864	Yan Cheng	0.969	Hu Zhou	1.039
22	Chi Zhou	0.788	Zhen Jiang	0.637	Jin Hua	0.947
23	Jin Hua	0.778	Chi Zhou	0.514	Xuan Cheng	0.713
24	Yan Cheng	0.717	Jin Hua	0.347	Chi Zhou	0.507
25	Tai Zhou	0.646	Tong Ling	0.316	Tai Zhou	0.205
26	Zhou Shan	0.162	Zhou Shan	0.296	Zhou Shan	0.000
