# Supplementary material for: Characteristics and evolution of knowledge innovation network in the Yangtze River Delta urban agglomeration——A case study of China National Knowledge Infrastructure
Source: PLoS One. 2023 Apr 21;18(4):e0283853. doi: 10.1371/journal.pone.0283853 (PMC10121040; doi:10.1371/journal.pone.0283853)
Supplement: S3 Table — (PDF) [file pone.0283853.s003.pdf]

S3 Table.

Ranking	City	2010	City	2015	City	2020
1	Shang Hai	25	Shang Hai	25	Shang Hai	25
2	Nan Jing	25	Nan Jing	25	Nan Jing	25
3	Su Zhou	26	Su Zhou	26	Su Zhou	25
4	Chang Zhou	30	Ning Bo	28	Hang Zhou	25
5	Ning Bo	30	Wu Xi	30	Wu Xi	26
6	Hang Zhou	30	He Fei	30	He Fei	26
7	Wu Xi	31	Chang Zhou	31	Chang Zhou	26
8	He Fei	31	Yang Zhou	31	Ning Bo	26
9	Yang Zhou	32	Hang Zhou	31	Yang Zhou	27
10	Jia Xing	34	Nan Tong	32	Wu Hu	29
11	Nan Tong	34	Shao Xing	32	Jia Xing	31
12	Shao Xing	35	Jia Xing	33	Ma An Shan	31
13	Hu Zhou	35	Tai Zhou	34	Chu Zhou	31
14	An Qing	36	Ma An Shan	34	An Qing	33
15	Tong Ling	37	Wu Hu	34	Hu Zhou	33
16	Tai Zhou	37	An Qing	35	Jin Hua	33
17	Jin Hua	38	Chu Zhou	35	Yan Cheng	33
18	Wu Hu	38	Hu Zhou	36	Zhen Jiang	33
19	Yan Cheng	38	Yan Cheng	36	Nan Tong	33
20	Zhen Jiang	38	Tai Zhou	36	Shao Xing	33
21	Zhou Shan	40	Xuan Cheng	36	Tai Zhou	33
22	Ma An Shan	40	Zhen Jiang	37	Tong Ling	34
23	Tai Zhou	40	Tong Ling	38	Xuan Cheng	35
24	Chu Zhou	40	Jin Hua	39	Chi Zhou	36
25	Chi Zhou	41	Chi Zhou	39	Zhou Shan	37
26	Xuan Cheng	41	Zhou Shan	41	Tai Zhou	37
